# A commentary on ‘Effect of preoperative radiotherapy on stage IB2 and IIA2 cervical cancer: A retrospective cohort study’

**DOI:** 10.1097/JS9.0000000000001314

**Published:** 2024-03-18

**Authors:** Siyu Wu, Xiaoyu Yue, Yaping Wang

**Affiliations:** Department of Gynecology and Obstetrics, Qilu Hospital (Qingdao), Cheeloo College of Medicine, Shandong University, 266000 Qingdao, People’s Republic of China

It was with great interest that we read the article published in the *International Journal of Surgery*. Zhang *et al*.^1^ conducted a retrospective study on preoperative intracavitary radiotherapy combined with radical surgery in patients with stage IB2 and IIA2 cervical cancer (CC). The findings showed that radical surgery plus preoperative irradiation could raise the rate of locoregional control without raising the risk of surgical complications.

CC is a common malignant tumor of the female genital tract that gravely endangers women’s health. Approximately 560 000 new instances of CC are identified worldwide each year, with developing nations accounting for 80% of these cases. Early cervical malignancies have traditionally been treated with surgery, whereas advanced cases have been treated with chemotherapy and radiation. Regarding the best course of action for cervical tumors at stage IB2, which lies between early and advanced illness, there remains, still, disagreement. Chemoradiotherapy (CCRT), radical surgery (RS), radical surgery after chemoradiotherapy (CCRT+RS), neoadjuvant chemotherapy followed by radical surgery (NACT+RS), adjuvant radiotherapy followed by radical surgery (RT+RS), and radiotherapy alone (RT) are the mainstays of treatment for stage IB2/IIA2 CC. According to the findings of a recent study, individuals with 2009 FIGO (International Federation of Gynecology and Obstetrics) stage IB2/IIA2 CC had comparable therapy options, including primary surgical treatment (PST), NACT+RS, and CCRT^2^. The results of Zhou *et al*.^3^ demonstrated that RS combined with adjuvant therapy is a workable and successful treatment for IB2/IIA2 CC, with a respectable incidence of comorbidities and a good 5-year survival rate. A Bayesian network meta-analysis revealed that CCRT would be the most effective strategy for enhancing the clinical outcome of patients with CC diagnosed at FIGO (2009) stage IB2/IIA2^4^.

This research looks at the potential benefits of both urgent radical surgery and preoperative radiation. This creative approach provides enlightening guidance and inspiration for the continued development of this area of research. Still, there were a lot of limitations. First, there may be some selection bias in this single-center retrospective investigation. Secondly, bias resulted from the absence of some clinical features, such as a past illness. In addition to the demographic information and clinical factors gathered, information on pretreatment hemoglobin, lymph node metastases, vascular tumor thrombus, vaginal margin, deep stromal penetration, and any adjuvant treatment of patients should be provided. Ultimately, it can be challenging to monitor long-term consequences in retrospective research, which means that the analysis’s rates might not match the real rates.

Which of the current treatment choices (chemoradiotherapy or major surgery) is superior for stage IB2 CC is not known with great certainty at this time^5^. Women with stage IB2 CC should get counseling regarding this uncertainty and possible adverse effects. The patient’s preference and the treatments’ accessibility within a certain health resource should be considered when selecting a course of treatment. Prospective multicenter randomized controlled trials with large sample sizes are still needed to fully investigate the clinical outcomes of patients who may eventually receive preoperative intracavitary radiation in addition to radical surgery. Subgroup analysis and long-term follow-up will be required if more cases are added later.

## Ethical approval

This manuscript is a comment. Don’t need ethical approval.

## Consent

This manuscript is a comment. Don’t need patient consent.

## Sources of funding

This work was funded by the hospital foundation (QDKY2023BS15).

## Author contribution

S.W.: study concept or design, data collection, data analysis or interpretation, and writing the paper; X.Y.: data analysis or interpretation; Y.W.: study concept or design, writing, and revising the paper.

## Conflicts of interest disclosure

This manuscript is a comment without conflicts of interest.

## Research registration unique identifying number (UIN)

This manuscript is a comment. Don’t need UIN.

## Guarantor

Yaping Wang.

## Data availability statement

This manuscript is a comment. Don’t need a Data availability statement. However, all the data from the current study are publicly available.

## Provenance and peer review

This manuscript is a comment without being invited.
